# Involvement of Neutrophil Hyporesponse and the Role of Toll-Like Receptors in Human Immunodeficiency Virus 1 Protection

**DOI:** 10.1371/journal.pone.0119844

**Published:** 2015-03-18

**Authors:** Juan C. Hernandez, Diana M. Giraldo, Stephane Paul, Silvio Urcuqui-Inchima

**Affiliations:** 1 INFETTARE, Facultad de Medicina, Universidad Cooperativa de Colombia, Medellin, Colombia; 2 Grupo Inmunovirología, Facultad de Medicina, Universidad de Antioquia, Medellín, Colombia; 3 GIMAP EA3064, Faculté de Medicine de Saint Etienne, Université de Lyon, Lyon, France; Dasman Diabetes Institute, KUWAIT

## Abstract

**Objectives:**

Neutrophils contribute to pathogen clearance through pattern recognition receptors (PRRs) activation. However, the role of PRRs in neutrophils in both HIV-1-infected [HIV-1(+)] and HIV-1-exposed seronegative individuals (HESN) is unknown. Here, a study was carried out to evaluate the level of PRR mRNAs and cytokines produced after activation of neutrophils from HIV-1(+), HESN and healthy donors.

**Methods:**

The neutrophils were stimulated with specific agonists for TLR2, TLR4 and TLR9 in the presence of HIV-1 particles. Pro-inflammatory cytokine production, expression of neutrophil activation markers and reactive oxygen species (ROS) production were analyzed in neutrophils from HESN, HIV-1(+) and healthy donors (controls).

**Results:**

We found that neutrophils from HESN presented reduced expression of PRR mRNAs (TLR4, TLR9, NOD1, NOD2, NLRC4 and RIG-I) and reduced expression of cytokine mRNAs (IL-1β, IL-6, IL-18, TNF-α and TGF-β). Moreover, neutrophils from HESN were less sensitive to stimulation through TLR4. Furthermore, neutrophils from HESN challenged with HIV-1 and stimulated with TLR2 and TLR4 agonists, produced significantly lower levels of reactive oxygen species, versus HIV-1(+).

**Conclusions:**

A differential pattern of PRR expression and release of innate immune factors in neutrophils from HESN is evident. Our results suggest that lower neutrophil activation can be involved in protection against HIV-1 infection.

## Introduction

From the earliest days of HIV-1 epidemic, reports have described individuals who are HIV-1-exposed seronegative (HESN) [1]. Early studies proposed that these individuals exhibit an immunological advantage that provides a natural resistance against HIV-1 infection [2, 3]. Natural resistance to most infections could be mediated mainly by the innate immunity system. It comprises a variety of cell types responsible for the recognition of pathogens through a diverse array of pattern-recognition receptors (PRRs) [4]. Neutrophils play a major role in the innate immune response to pathogens; these are massively recruited to the sites of inflammation and sense microbial products through PRRs [5, 6]. However, although PRRs have a functional significance in neutrophils, studies aimed to defining the role of neutrophils in controlling the early stages of HIV-1 infection remain inconclusive. Indeed, neutrophil activation via Toll-like receptors (TLRs) resulted in the shedding of L-selecting (CD62L) and the up-regulation of CD11b, in a dose-dependent manner [7]. In addition, the induction of reactive oxygen species (ROS)- after TLRs stimulation in neutrophils has been reported [8]. Indeed, neutrophils recognize HIV-1 through TLR7/8 and produce ROS [9], which is associated with cell activation and secretion of pro-inflammatory cytokines, favoring HIV-1 replication. A similar effect was previously reported in human microglial cells and in HIV-1-latently infected promonocytic cells as well [10, 11]. Although stimulation of TLRs induced the activation of the NF-κB pathway [5], there is also strong evidence that in neutrophils, this cascade is amplified by the generation of ROS [12]. Despite the evidence showing that PRRs play an important role in the recognition of HIV-1, no studies focus on the function of neutrophils in HIV-1 infection processes and even less on the role of PRRs in neutrophils from HESN. Therefore, we assessed PRR expression and response in neutrophils from HIV-1(+), HESN and healthy donors (controls). Here, we report that expression and function of PRRs in neutrophils are clearly different. Indeed, purified neutrophils from HESN express lower levels of mRNAs for diverse PRR and cytokines, compared to HIV-1(+). Further, neutrophils from HESN produce significantly lower levels of IL-6 and higher levels of IL-10, in response to LPS. Finally, neutrophils from HESN challenged with HIV-1 and stimulated with Pam_2_CSK_4_ or LPS, produce significantly less ROS compared to HIV-1-infected patients. We propose that neutrophils may play an important role in the pathogenesis of HIV-1 infection.

## Materials and Methods

### Description of the cohort

To examine the function of TLRs in neutrophils (cytokine secretion, neutrophil activation markers and ROS production), we included four HIV-1(+) with their corresponding HESN partners, and eight controls. The mRNA expression of PRR, pro- and anti-inflammatory cytokines was evaluated in 9 HIV-1(+), 6 HESN and 12 controls (Table 1).

**Table 1:** Demographic features of HIV-1 infected patients, exposed seronegative donors and healthy controls.

	HIV-1-infected patients n = 9	Exposed seronegative donors (ESN) n = 6	Healthy Controls n = 12
Age Median (Range)	38 (28–47)	38 (29–50)	28 (21–40)
Male: Female	6: 3	2: 4	5: 7
Viral load in RNA copies/ml plasma Median (Range)	63700 (1261–109147)	N/A	N/A
With HAART^a^: Without HAART	5: 4	N/A	N/A
CD4+ T-cells count cells/μl peripheral blood^b^ Median (Range)	304 (14–948)	997 (977–1433)	710 (617–1143)

^a^Patients in HAART treatment were using combinations of nucleoside reverse transcriptase inhibitors (abacavir, lamivudine, didanosine, stavudine, and zidovudine), nonnucleoside reverse transcriptase inhibitors (efavirenz and nevirapine), and protease inhibitors (lopinavir, fosamprenavir, amprenavir, nelfinavir, and saquinavir).

^b^CD4+ T cell counts under 200 cells/ll, were receiving fluconazol, acliclovir, and TMS as prophylactic drugs.

HAART, highly active antiretroviral therapy; N/A, not applicable

Inclusion criteria of HESN were a history of multiple unprotected penetrative sexual intercourses with an HIV-1-infected individual more than five times in the previous 6 months, or an average of twice weekly for over 4 months within the last 2 years, and a negative HIV-1/2 ELISA test within one month before sampling [13, 14]. None of the HESN had a history of intravenous drug use, or active disease; HIV-1 infection was confirmed by western blot. HIV-1 diagnostics were performed as previously described [15]. HIV-1(+) patients were negative for active opportunistic diseases. The study was designed and performed according to the Declaration of Helsinki and approved by the Ethics Committee (Universidad de Antioquia). All participants provided written informed consent to participate in this study.

### Neutrophil isolation

Neutrophil purification was performed as previously described [16], with modifications. Briefly, peripheral blood was obtained from HIV-1(+), HESN and controls, by venipuncture into tubes containing ACD anti-coagulant (BD Pharmingen, San Diego, CA). The red cells were precipitated by adding dextran (Sigma-Aldrich Chemical Co., St. Louis, MO) in 0.9 M NaCl and incubating 50 min at 4°C, and the upper phase which corresponds to leukocyte-rich plasma was collected. The pellet underwent lysis of the remaining red blood cells followed by density gradient centrifugation with Ficoll-Hypaque 1077 (Sigma-Aldrich Chemical Co.). The neutrophils were re-suspended in RPMI1640 (BioWhittaker, Walkersville, MD) enriched with 10% heat-inactivated fetal bovine serum (FBS). This protocol yielded neutrophils that were 94–96% pure, 98–99% viable and without basal activation, measured by IL-6 production (data not shown).

### Preparation of virus stocks

HIV-1 stock was collected from chronically HIV-1-infected H9-HTLV-IIIcc cells (NIH AIDS Research & Reference Reagent Program) and prepared as previously described [17].

### *In vitro* HIV-1 stimulation study

Neutrophils were cultured at 1.25x10^6^ cells/ml in 96-well polystyrene tissue culture plates at 37°C and 5% CO_2_, using FBS-supplemented RPMI1640 medium. Neutrophils were then challenged with HIV-1_H9-HTLV-IIIcc_ (5 ng p24/ml), during 8 h for functional analyses. Further assays challenged the neutrophils with HIV-1 in the presence of 20 ng/ml of Pam_2_CSK_4_ or 0.1 ng/ml of ultrapure LPS from *Escherichia coli* 0127:B8 (Invivogen, San Diego, CA). The cells and supernatants were harvested after 8 h of culture and assayed for TNF-α, IL-6, IL-10 and IL-1β release, CD62L and CD11b cell-membrane expression and ROS production.

### Monoclonal antibodies

Monoclonal antibodies for CD11b-PE-Cy5 (clone ICRF44) and CD62L FITC (clone DREG-56) were from BD Biosciences, and monoclonal antibodies for TLR2 PE (clone TL2.1) and TLR4 PE (clone HTA125) were from eBiosciences (San Diego, CA). FcR blocking reagent was from Miltenyi Biotec, Auburn, CA. Conjugated isotype-control antibodies served as controls.

### Flow cytometry analysis

Flow cytometry was used to evaluate the effect of HIV-1 infection on the expression of TLR2, TLR4, CD62L and CD11b on neutrophils. For this purpose, freshly isolated neutrophils were surface-stained with the appropriate antibodies for 25 min; the acquisition was performed immediately using a FACSCan flow cytometer (BD Biosciences, San Jose, CA). The acquired events were analysed using FACS Diva software version 6.1.2. Receptor expression is expressed as the mean fluorescent intensity (MFI) of the overall cell sub-population after subtraction of the isotype control.

### RNA isolation, cDNA synthesis and quantitative real-time PCR for PRR and cytokine mRNAs

mRNA quantification for cytokines (IL-6, TNF-α, IL-1β, IL-18 and TGF-β) and PRR (TLR2, TLR4, TLR7, TLR8, TLR9, RIG-I, MDA-5, NOD1, NOD2, NLRP1 and NLRC4) was performed in neutrophils from HIV-1(+), HESN and controls. For total RNA preparation, the RNeasy mini isolation kit was used (Qiagen, Valencia, CA). cDNA synthesis and quantitative real-time PCR for PRR and cytokine mRNAs were performed as previously described [15, 17]. Primer sequences are listed in S1 Table.

### ELISA

IL-6, TNF-α, IL-1β and IL-10 were quantified by ELISA (BD Biosciences) as reported [17].

### Quantification of ROS

The production of ROS was quantified using dihydrorhodamine123 (DHR123), a lipophilic oxidation-sensitive indicator of ROS (Invitrogen, San Diego, CA), in neutrophils challenged with HIV-1 for 2 h. Briefly, DHR123 was diluted 1:2000 and incubated 8 h at 37°C; the neutrophils were then collected and ROS formation in neutrophils was measured immediately by monitoring ROS-mediated conversion of DHR123 to fluorescent rhodamine 123, using a FACSCan flow cytometer (BD Biosciences).

### Statistical analysis

Data were plotted and analyzed using the Prism 5.0 software (Graph Pad Software, CA). The results of the *in vitro* assays represent all the data collected from patients, cultured in duplicates. Unpaired two-tailed student t-tests (Mann–Whitney U-tests) and ANOVA tests were used to assess the statistical significance of PRR expression or function in the different groups, when data from more than three independent experiments were available. Values of *p*<0.05 (*) and *p*<0.01 (**) were considered statistically significant.

## Results

### Absence of neutropenia in HESN individuals

Early asymptomatic HIV-1 infection and advanced HIV-1-related immunodeficiency are associated with neutropenia [18]. No significant differences were observed when the number or percentage of neutrophils was evaluated among the groups of donors.(data not shown). Moreover, HESN have significantly higher CD4^+^ T-cell, mDC and pDC counts compared with HIV-1(+) (data not shown).

### HESN-derived neutrophils have a low inflammatory profile after TLR stimulation

**Fig 1:**
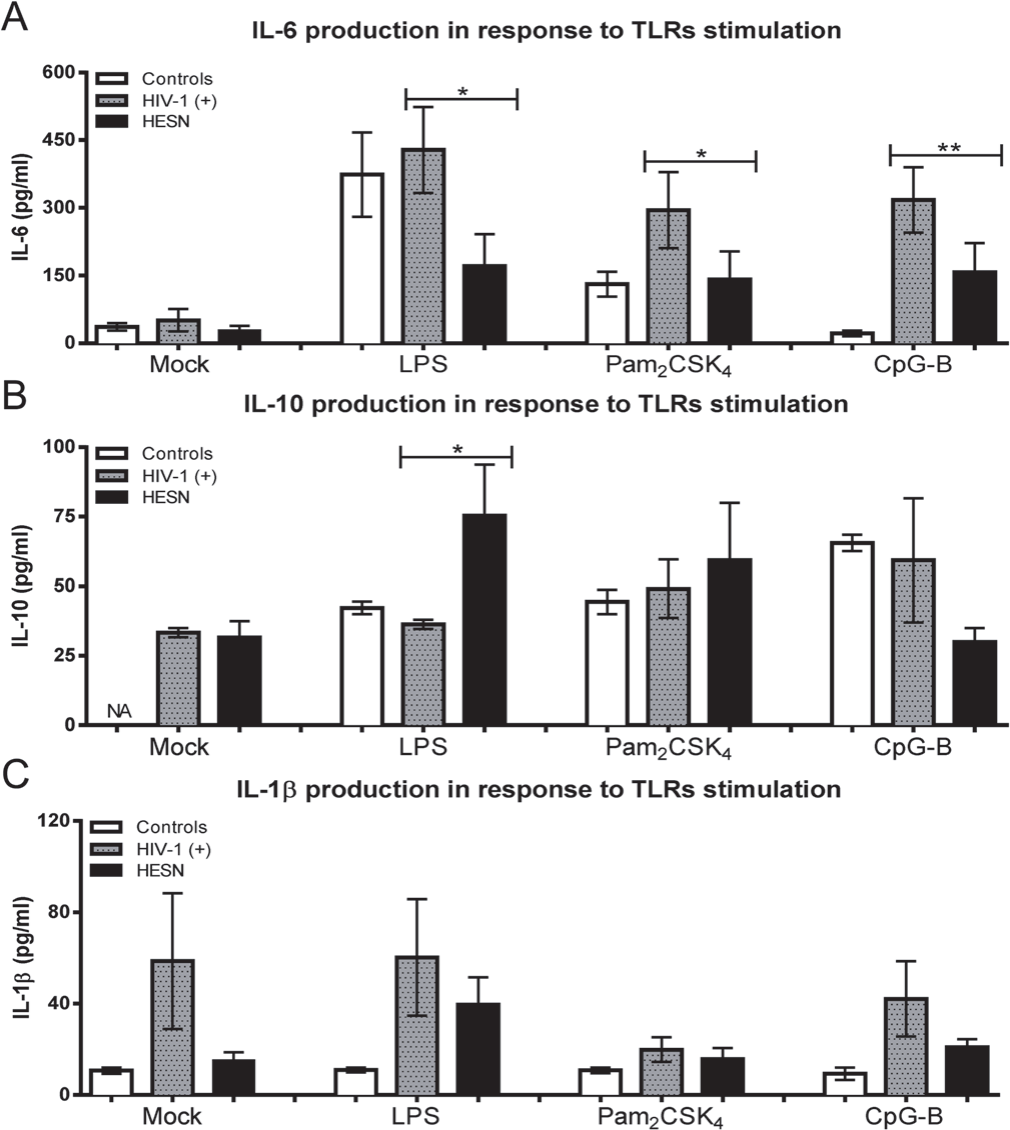
Decrease of IL-6 and increase of IL-10 secretion in HESN derived-neutrophils after TLR4 stimulation. Neutrophils were purified and treated with TLR2 (20 nM Pam_2_CSK_4_), TLR4 (0.1 ng/ml LPS) or TLR9 (10 μM CpG-B) agonists for 18 h at 37°C and 5% CO_2_. Then (A) IL-6, (B) IL-10 and (C) IL-1β secretion were evaluated in the supernatants by ELISA. Comparisons were performed using the Kruskal-Wallis ANOVA tests and Dunn’s post-tests. The levels of significance were p<0.05 (*) and p<0.01 (**).

To determine whether TLR-triggering leads to cytokine secretion, the pro- and anti-inflammatory cytokine profiles following TLR stimulation were studied. Activation of TLR2, TLR4 and TLR9 induced a significant decrease of IL-6 release in neutrophils from HESN versus those from HIV-1(+) (Fig. 1A). In addition, neutrophils from HESN express significantly higher levels of IL-10 in response only to LPS (Fig. 1B). When IL-1β was quantified, no statistical differences were observed among the groups; however the trend has been towards a slight decrease for TLR4 and TLR9 stimulation (Fig. 1C).

### Significantly reduced expression of PRRs in neutrophils from HESN

**Fig 2:**
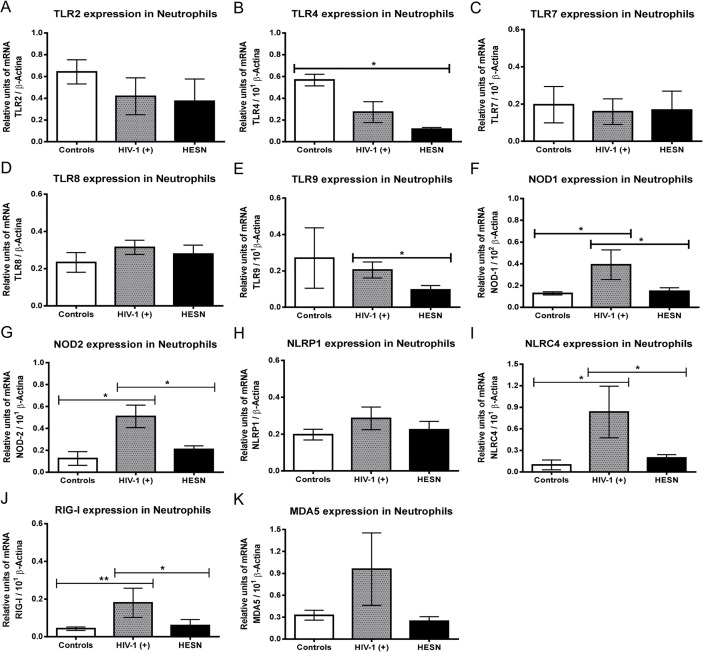
Reduced expression of TLRs, NLRs and RLRs in HESN neutrophils. Neutrophils were purified from each of the three populations under investigation and the PRR mRNAs were quantified using quantitative real time RT-PCR, and normalized with the housekeeping gene β-actin. Relative units of transcripts versus housekeeping gene transcripts are shown as median and range. Comparisons were by the Kruskal-Wallis ANOVA test and Dunn’s post-test. The levels of significance were p<0.05 (*) and p<0.01 (**).

Neutrophils belong to the first line of defense against pathogens, and are prime candidates as virus sensors through PRRs. Nevertheless, the expression of PRRs has not been evaluated in neutrophils from HIV-1(+) and HESN. Based on our results, it is clear that *in vitro* stimulation with HIV-1 or its genome alters the expression and function of TLRs and RIG-I-like receptors of neutrophils (manuscript in preparation). Thus, expression of various PRRs was measured in neutrophils. Significantly reduced mRNA expression of TLR9, NOD1, NOD2, NLRC4 and RIG-I in neutrophils from HESN was observed (Fig. 2E-G, I-J), compared with those from HIV-1(+). The mRNA expression of TLR4 in neutrophils from HESN was also significantly reduced compared with those from controls (Fig. 2B). The mRNA expression of NOD1, NOD2, NLRC4 and RIG-I was significantly increased in neutrophils from HIV-1(+) compared with those from controls (Fig. 2F-G, I-J). In accordance withTLR2 and TLR4 mRNA expression, quantification at the protein level also shows a decreased expression of TLR2 and TLR4 in neutrophils from HESN compared to healthy donors or HIV-1-infected patients, respectively (S1 Fig.). Overall, these data indicate that selected PRRs involved in innate sensing pathways were significantly reduced in neutrophils from HESN compared with HIV-1(+) and suggest that neutrophils can sense an array of microbial compounds *via* a broad repertoire of PRRs. This behavior could be very important for resistance to both HIV-1 infection and HIV-1 immunopathogenesis.

### Reduced expression of pro- and anti-inflammatory cytokines in neutrophils from HESN

**Fig 3:**
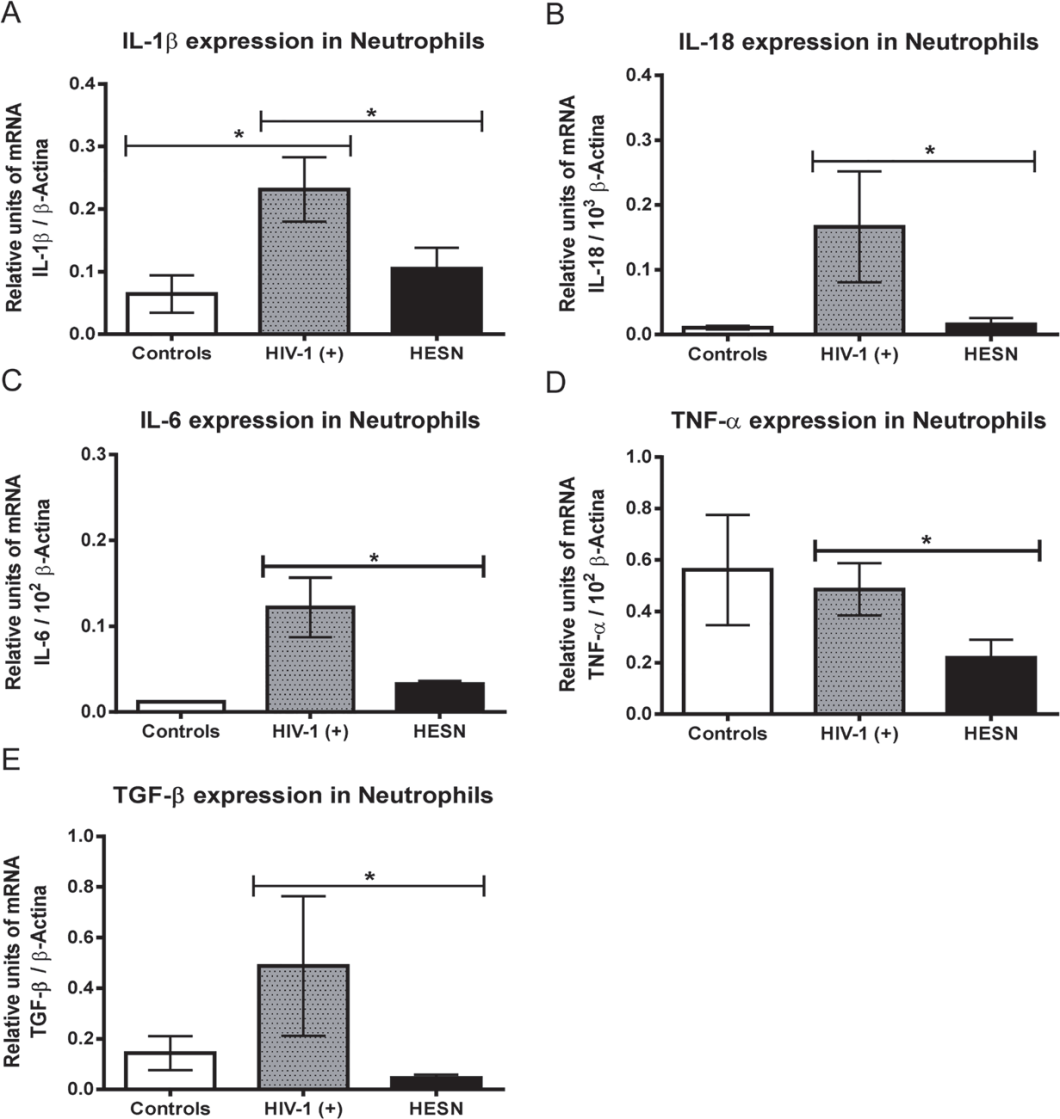
Reduced expression of pro- and anti-inflammatory cytokines in neutrophils of HESN individuals. Neutrophils were purified from each of the three populations under study and the cytokine mRNAs (IL-1β, IL-18, IL-6, TNF-α and TGF-β) were quantified using quantitative real time RT-PCR, and normalized with the housekeeping gene β-actin. Relative units of transcripts versus housekeeping gene transcript are shown as median and range. Comparisons were by the Kruskal-Wallis ANOVA test and Dunn’s post-test. The level of significance was p<0.05 (*).

Since neutrophils from HESN express lower levels of PRRs, we determined whether these neutrophils were also immunologically dysfunctional. First, mRNAs extracted from neutrophils at a steady state, were tested by quantitative RT-PCR, for the expression of IL-1β, IL-6, IL-18, TNF-α and TGF-β. HESN-derived neutrophils contained significantly lower levels of mRNAs corresponding to these cytokines versus those from HIV-1(+) (Fig. 3A-E). Interestingly, neutrophils from HESN and controls contained a similar lower amount of IL-1β mRNA versus those from HIV-1(+) (Fig. 3A). These results suggest that significantly lower mRNA expression levels of PRRs could be linked with reduced mRNA expression of pro- and anti-inflammatory cytokines. This could be protective in HESN, in terms of impaired immune activation in response to HIV-1 infection. It could suggest that the immune-quiescent phenotype may be linked to preventing inflammation and protecting this group of individuals against HIV-1 infection.

### Differential modulation of neutrophil activation markers in response to TLR stimulation and HIV-1 challenge

Neutrophil stimulation through TLRs also promotes the production of adhesion molecules, including CD11b and CD62L [7]. Expression of adhesion molecules correlates with the activation status of neutrophils. Since neutrophils from HESN have lower expression of PRRs and cytokines, the function of TLRs was determined. Therefore, CD11b and CD62L expression in freshly purified neutrophils from HIV-1(+), HESN and controls was determined after stimulation with Pam_2_CSK_4_, LPS and R848, agonists of TLR2, TLR4 and TLR7/8, respectively, and in the presence or absence of HIV-1.

**Fig 4:**
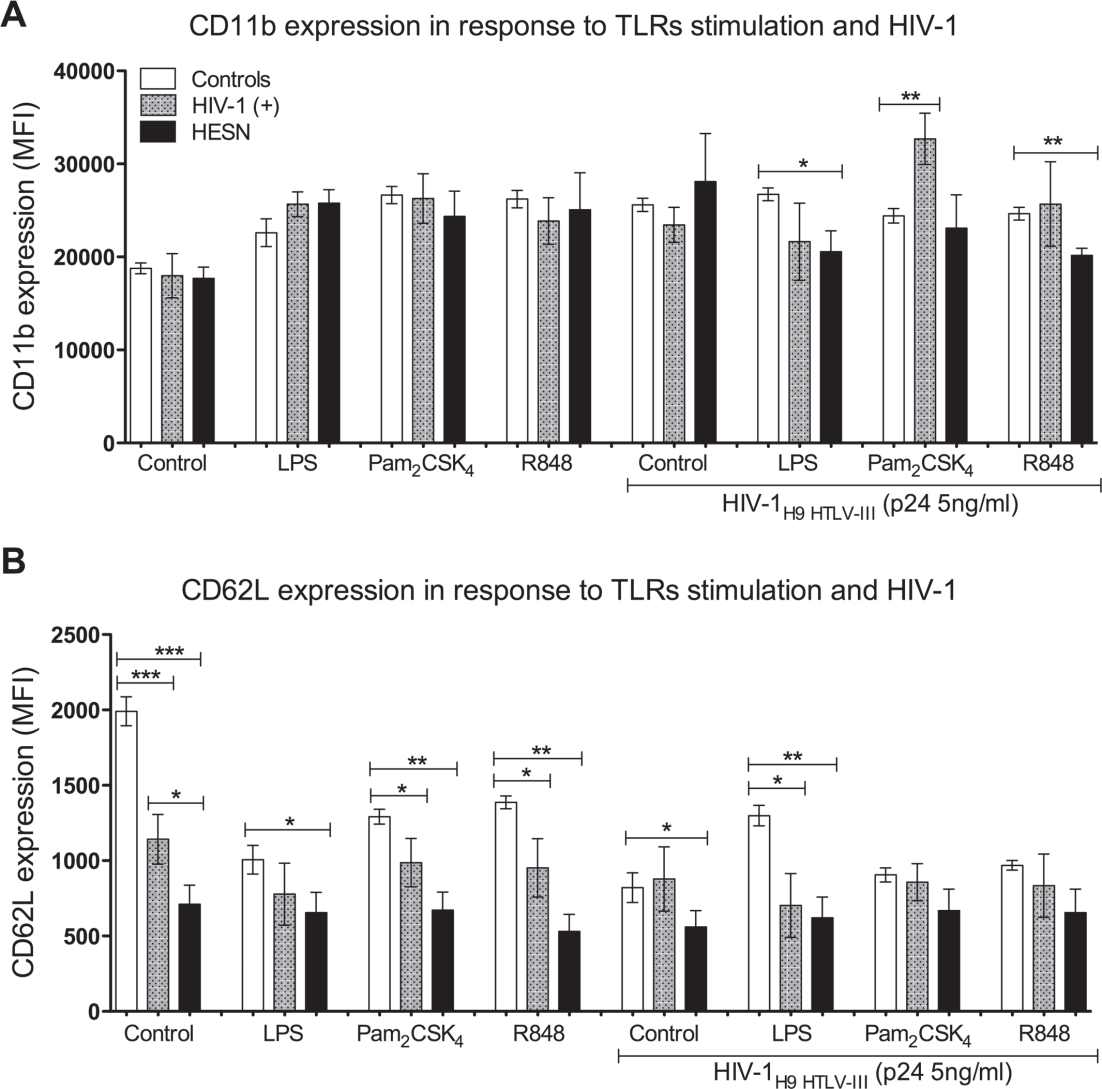
Expression of activation markers in neutrophils from HESN after co-stimulation with TLR agonists and HIV-1. After purification, the neutrophils were stimulated with TLR2, TLR4 or TLR7/8 agonists in the presence or absence of HIV-1. The CD11b (A) and CD62L (B) markers were quantified by flow cytometry. Neutrophils were gated according to physical characteristics, excluding dead cells. Data are presented as overall MFI, after subtraction of isotype staining background. Comparisons were performed using the Kruskal-Wallis ANOVA test and Dunn’s post-test. The levels of significance were p<0.05 (*), p<0.01 (**) and p<0.001 (***).

There was no difference in the basal expression level of CD11b, between neutrophils from HIV-1(+), HESN and controls (Fig. 4A) but a trend was noted towards an increase of CD11b expression in neutrophils in the three populations studied after stimulation by TLR agonists (Fig. 4A). However, the MFI for CD11b in neutrophils from HESN was considerably decreased after co-stimulation with LPS or R848 in the presence of HIV-1 particles, compared to controls (Fig. 4A). Neutrophils from HIV-1(+) significantly increased the expression of CD11b when co-stimulated with HIV-1 and TLR2 agonist, compared to controls (Fig. 4A). This suggests an alteration of neutrophil activation by HIV-1 in the presence of some TLR agonists.

Next, we evaluated the expression of CD62L. A significantly higher expression of CD62L was observed in neutrophils from controls, compared to those from HIV-1(+) and HESN (Fig. 4B) at the basal level, indicating cellular activation in HIV-1(+) and HESN neutrophils. A significantly lower expression of CD62L was also observed in neutrophils from HESN stimulated with Pam_2_CSK_4_, LPS and R848, compared with those from controls (Fig. 4B). A similar result was also observed in neutrophils from HIV-1(+), but only in response to Pam_2_CSK_4_ and R848, versus those from controls. Stimulation with HIV-1 particles led to considerable reduction of CD62L expression, but only in neutrophils from HESN, compared with those from controls. Finally, the expression level of CD62L was much lower in neutrophils from HESN and HIV-1(+), compared with controls, but only in response to co-stimulation with LPS and HIV-1 (Fig. 4B). These results suggest that neutrophils from HIV-1(+), HESN and controls, respond differently to stimulation with HIV-1 and TLR agonists.

### Decrease of ROS production in neutrophils from HESN

**Fig 5:**
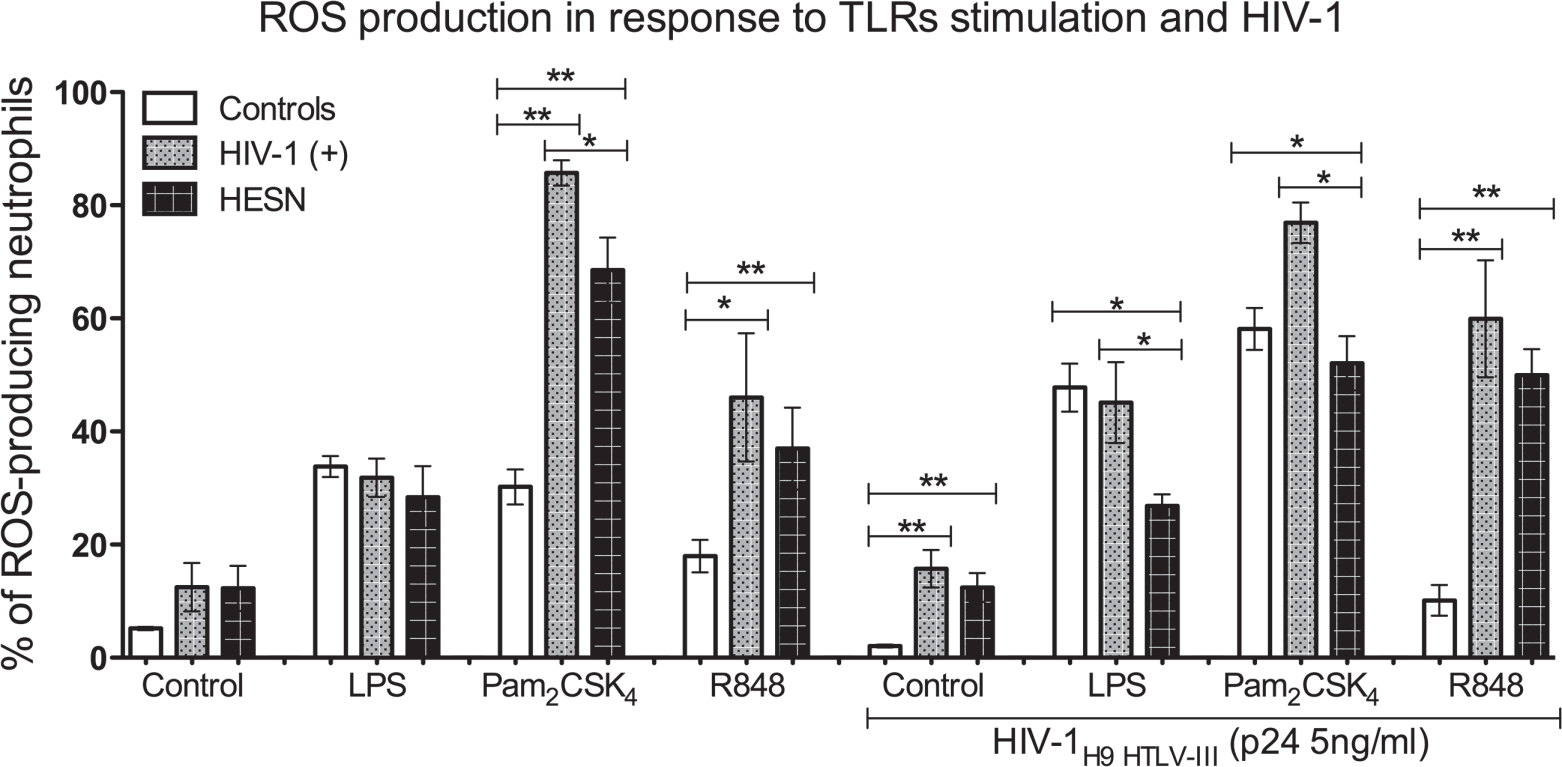
Reduced expression of ROS in neutrophils of HESN individuals co-stimulated with TLR2 and TLR4 agonists and HIV-1. After purification, the neutrophils were stimulated with TLR2, TLR4 or TLR7/8 agonists in the presence or absence of HIV-1 and ROS was quantified by flow cytometry. Neutrophils were gated according to physical characteristics, excluding dead cells. Data are presented as percentage of ROS-producing neutrophils, after subtraction of background. Comparisons were performed using the Kruskal-Wallis ANOVA test and Dunn’s post-test. The levels of significance were p<0.05 (*) and p<0.01 (**).

Among phenotypic characteristics of neutrophils, ROS production may constitute a hallmark of neutrophil function, as an antimicrobial mechanism against invading pathogens. Since the effect of HIV-1 and TLR agonists on neutrophil ROS production is not well known, we probed the outcome of co-stimulation with TLR agonists and HIV-1 on intracellular ROS production in neutrophils. At basal levels, neutrophils from all groups of donors produce low levels of ROS (Fig. 5). Nevertheless, a statistically significant increase in ROS production was observed in neutrophils from HESN and HIV-1(+) compared with those from controls, in response to stimulation with TLR2 and TLR7/8 agonists (Fig. 5). However, neutrophils from HESN produced considerably lower levels of ROS than those from HIV-1(+) in response to the TLR2 agonist. TLR4 stimulation with LPS had similar effects on ROS production. Interestingly, in response to HIV-1 and TLR4 co-stimulation, a statistically significant decrease in ROS production occurred in neutrophils from HESN, compared with those from HIV-1(+) and controls (Fig. 5). In contrast, neutrophils from HIV-1(+) produced higher levels of ROS, in response to co-stimulation with HIV-1 and TLR2 agonists versus those from HESN and controls (Fig. 5). Finally, in the presence of the TLR7/8 agonist and HIV-1, intracellular ROS production was induced in neutrophils from HIV-1(+) and HESN compared to controls (Fig. 5). Consequently, neutrophils from HESN produce lower levels of ROS in response to TLR2/TLR4 agonists, than those from HIV-1-infected individuals and this could have a potential effect on neutrophil functions against HIV-1.

## Discussion

The functions of neutrophils from HIV-1(+) and HESN have so far not been addressed. Here, we describe the transcription level of a wide number of PRRs and cytokines in primary neutrophils from HIV-1(+), HESN and controls. Indeed, in neutrophils from HESN, lower levels of mRNAs were detected for TLR9, NOD1, NOD2, NLRC4, and RIG-I compared with HIV-1(+). Our data agree with previous results showing significantly lower levels of innate immune activation in blood and lymph nodes from chronically SIV-infected non-human primates compared to pathogenic SIV-infection, including RIG-I and MDA-5[19]. Additionally, it was also demonstrated that cervical mononuclear cells and epithelial cells from HESN differentially expressed innate PRRs compared with HIV-1(+) [20]. A decreased immune activation was also demonstrated by microarray analysis in HESN [21].

Consistent with low levels of PRR mRNAs, we also found that neutrophils from HESN express significantly lower levels of IL-6 in response to TLR agonists, compared with HIV-1(+) and controls.TLR2 recognizes diacyl lipopeptides, such as Pam_2_CSK_4_ in combination with TLR6 [22]. Since IL-6 production after TLR2 stimulation was reduced in neutrophils from HENS versus HIV-1-infected patients and we did not observe a difference in TLR2 expression either at the protein or mRNA level, we speculated that IL-6 production in response to Pam_2_CSK_4_ can be affected by TLR6 expression, as previously reported by Sawahata et al. who showed that TLR6 helps to amplify the TLR2 signal by lipopeptides [23].

In contrast, IL-10 secretion was significantly increased in neutrophils from HESN, stimulated with TLR4 agonists, versus those from HIV-1(+) and controls (Fig. 1A and B). It was recently demonstrated that TLR2 agonists block HIV-1 replication through IL-10 and chemokine secretion [24]. Nevertheless, we recently reported an increase of TLR2/TLR4 expression and pro-inflammatory cytokines on monocytes-derived macrophages and PBMC subpopulations from controls after *in vitro* stimulation with HIV-1[17] and, in dendritic cells from HIV-1(+), without HAART, and co-infected with opportunistic infections [15].

Thus, and as previously proposed by other authors [20], our current results suggest that a balance in cytokine production may contribute to the maintenance of innate immune quiescence, and play an important role in controlling the excessive immune activation and inflammation that could favor susceptibility to HIV-1 infection.

Genetic variation in TLR4/TLR9 has been associated to host interaction with HIV-1 and rapid disease progression [25, 26], but the mechanisms whereby these TLRs modulate HIV-1 still remain unclear. Furthermore, neutrophils can detect HIV-1 particles via TLR7/8 [9]. Since TLRs are the main pathway in pro-inflammatory cytokine secretions, we propose that neutrophils may participate actively in the inflammatory response associated with HIV-1 pathogenesis. This is supported by the fact that circulating LPS and systemic immune activation are well correlated in HIV-1(+) [27] and that LPS in the bloodstream are indicative of microbial translocation across the gut mucosa [28]. Furthermore, the neutrophils from asymptomatic HIV-1(+) presented an exaggerated response to LPS [29]. Taken together, we can speculate that the microbiome may contribute to AIDS progression through neutrophil activation as observed in HIV-1(+). However, further studies are required to support our hypothesis.

Our results also indicate that primary neutrophils from HESN express low levels of NOD1, NOD2 and the inflammasome NLRC4, compared to HIV-1(+). NOD-like receptors are expressed in innate immune cells, such as neutrophils [30] and participate actively in sensing intracellular pathogens and in the induction of inflammatory responses [31]. The role of the inflammasome in HIV-1 infection or its antiviral activity against HIV-1 has to date not been demonstrated. The importance of the inflammasome mechanisms in recognizing HIV-1 particles has only recently emerged [32] and susceptibility to HIV-1 infection is associated with polymorphisms in inflammasome genes [33]. We recently reported that HIV-1 promotes the production of IL-1β by inducing the first signal of NLRP3 inflammasome activation [34]. It was recently demonstrated that NOD1 and NOD2 ligands induce a strong up-regulation of maturation markers in dendritic cells and enhance pro-inflammatory cytokine secretion [35]. Moreover, Ghosh *et al*., reported that primary epithelial cells from the human female reproductive tract exposed to *Neisseria gonorrhea* and HIV-1 increase the expression of NOD1, NOD2, RIG-I and MDA5 [36]. In addition, treatment of dendritic cells with agonists of dectin1, TLR2 and NOD2, renders them highly susceptible to HIV-1 infection and promotes *cis*-infection of autologous CD4^+^ T cells [37]. We also found a significant decrease in RIG-I mRNA level in neutrophils from HESN compared to those from HIV-1(+) (Fig. 2J). However, it is important to indicate that HIV-1 genomic RNA induces type I interferon production through RIG-I stimulation [38].

PRR stimulation is essential for cytokine production and immune activation. Neutrophils from HESN also have significantly lower levels of mRNAs for IL-1β, IL-6, IL-18, TNF-α and TGF-β than those from HIV-1(+) (Fig. 3). It is therefore, striking that these cytokines, which seem to be important in the establishment and progression of HIV-1 infection, were lower in neutrophils from HESN, suggesting a potential role in protection from HIV-1 infection. Low levels of these cytokines in neutrophils seem to trigger an alteration in their activation status, affecting the HIV-1 ability to bind neutrophils, since it was demonstrated that activation of neutrophils by inflammatory stimuli increases the binding of HIV-1 and their ability to infect PBMCs [39]. The low expression of anti- and pro-inflammatory cytokines, observed in primary neutrophils from HESN, could be associated with the low expression of PRRs, as previously reported in cervical-vaginal washes from HESN compared to HIV-1(+) [20]. This is of great importance as increased levels of genital pro-inflammatory cytokines and other immune factors have been associated with increased HIV-1 acquisition in women from sub-Saharan Africa [40, 41]. Previous studies have described reduced immune activation in lymphocytes from HESN [42–44]. More recently it was demonstrated that female HESN sex workers produce less pro-inflammatory Th17/Th22-type cytokines in cervical secretions [45], genital tract and blood [46]. Unstimulated PBMCs from HESN women were also reported with lower levels of IL-1β, IL-6 and TNF-α, a phenomenon associated with an immune quiescence hypothesis [42]. Interestingly, IL-1β and IL-6 enhance HIV-1 infection in monocytes and resting CD4+ T cells *in vitro* [47, 48]. Regarding TNF-α, activation of dendritic cells and migration to lymph nodes has been reported [49]. This phenomenon could be associated with transport of HIV-1 infectious particles to target T-cells in lymph nodes, called the “Trojan horse” model of HIV-1 trans-infection [50, 51]. Thus, lower levels of cytokines may reduce HIV-1 replication, and contribute to HI-1 resistance in HESN.

Many studies have described the profiles of neutrophil adhesion molecules in adults infected with HIV-1 [52, 53]. However there are no reports concerning the expression of adhesion molecules in neutrophils from HESN. Since we observed that HESN neutrophils express lower levels of CD11b and CD62L but neutrophils from HIV-1(+) showed a higher production of CD11b in response to TLR agonists and HIV-1 challenge, dysregulation of adhesion molecule expression may contribute to HIV-1 disease progression. Indeed, elevated levels of soluble CD62L were correlated with higher viral load in HIV-1-infected children [54].

In macrophages from HIV-1(+), an impaired oxidative burst response has been described[55]. Here, we found that ROS production in neutrophils from HESN co-stimulated with TLR agonists and HIV-1 is reduced compared to those from HIV-1-infected patients, comparable to previous studies demonstrating increased ROS production in monocytes and macrophages from HIV-1-infected individuals [56, 57]. Therefore, low production of ROS can also contribute to our hypothesis related to innate immune quiescence and resistance to HIV-1 infection, because ROS production is involved in both pro-inflammatory cytokine production and HIV-1 LTR activation via post-translational control of NF-κB [58] and up-regulation of CD11b expression [59]. Interestingly, a connection between ROS production, cytokine secretion and TLR activation has been described [60]. Since ROS is involved in eliminating pathogens, inflammation and tissue injury, dysregulation of ROS production may contribute to HIV-1 immunopathogenesis.

## Conclusion

We have shown that neutrophils from HESN have a distinct pattern of PRR expression, cytokine and ROS production and a lower expression of activator markers. These results could contribute to the immune quiescence phenotype observed in HESN. Thus, the low innate immune response against HIV-1 could be involved in the protection reported in HESN. However, future studies will delineate how the balance of pro- and anti-inflammatory cytokine secretion and PRR expression in the face of HIV-1 infection is mediated in critical cells of the innate immune response, including neutrophils.

## Supporting Information

S1 Fig Reduced expression of TLR2 and TLR4 in HESN neutrophils. Neutrophils were purified from each of the three populations under investigation, and gated according to physical characteristics, excluding dead cells. Then, TLR2 and TLR4 were quantified using flow cytometry. Data are presented as as median and range of overall mean fluorescence intensity (MFI), after substracting the isotype-staining background. Comparisons were by the Kruskal-Wallis ANOVA test and Dunn’s post-test. The levels of significance were p<0.05 (*) and p<0.001 (***).
(TIFF)

S1 Table Primers used for PRRs and cytokine amplification by real-time PCR analysis (DOCX)
