# Supplementary figures and images for: Involvement of Neutrophil Hyporesponse and the Role of Toll-Like Receptors in Human Immunodeficiency Virus 1 Protection
Source: PLoS One. 2015 Mar 18;10(3):e0119844. doi: 10.1371/journal.pone.0119844 (PMC4364960; doi:10.1371/journal.pone.0119844)

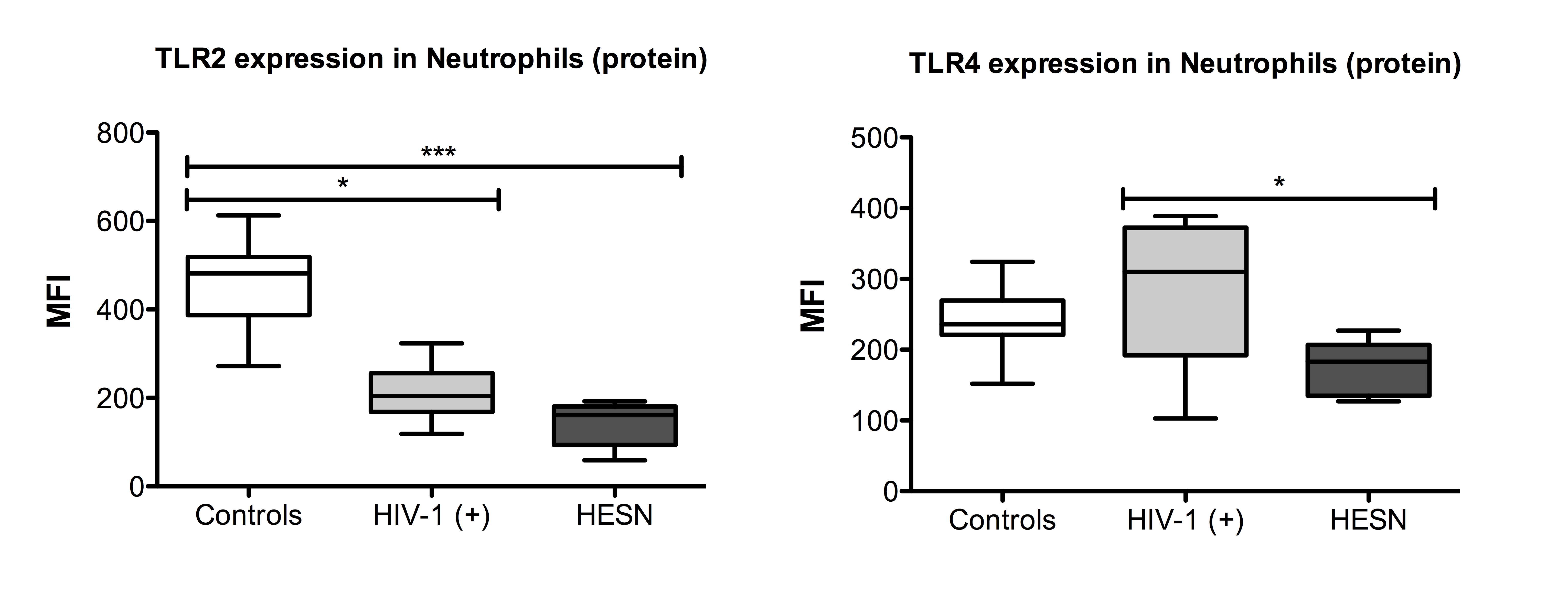

Supplement: S1 Fig — Neutrophils were purified from each of the three populations under investigation, and gated according to physical characteristics, excluding dead cells. Then, TLR2 and TLR4 were quantified using flow cytometry. Data are presented as as median and range of overall mean fluorescence intensity (MFI), after substracting the isotype-staining background. Comparisons were by the Kruskal-Wallis ANOVA test and Dunn’s post-test. The levels of significance were p<0.05 (*) and p<0.001 (***). (TIFF) [file pone.0119844.s001.tiff]
