# Supplementary material for: Involvement of Neutrophil Hyporesponse and the Role of Toll-Like Receptors in Human Immunodeficiency Virus 1 Protection
Source: PLoS One. 2015 Mar 18;10(3):e0119844. doi: 10.1371/journal.pone.0119844 (PMC4364960; doi:10.1371/journal.pone.0119844)
Supplement: S1 Table — (DOCX) [file pone.0119844.s002.docx]

**Table S1. Primers used for PRRs and cytokine amplification by real-time PCR analysis**

| **mRNA** | | **Primer 5’ 3’** |
| --- | --- | --- |
| **β-actin** | Forward | ATCTGGCACCACACCTTCTACAATGA |
|  | Reverse | CGTCATACTCCTGCTTGCTGATCCAC |
| **TLR2** | Forward | GCTGCTCGGCGTTCTCTCAGG |
|  | Reverse | TGTCCAGTGCTTCAACCCACAACT |
| **TLR4** | Forward | GCCCTGCGTGGAGGTGGTTCCTA |
|  | Reverse | AGCTGCCTAAATGCCTCAGGGGAT |
| **TLR7** | Forward | TCTACCTGGGCCAAAACTGTT |
|  | Reverse | GGCACATGCTGAAGAGAGTTA |
| **TLR8** | Forward | TGGGATCCGGGATTGGCCATCA |
|  | Reverse | ACAGATCCGCTGCCGTAGCCT |
| **TLR9** | Forward | TTATGGACTTCCTGCTGGAGGTGC |
|  | Reverse | CTGCGTTTTGTCGAAGACCA |
| **NOD1** | Forward | CCTAGACAACAACAATCTCAACGACTA |
|  | Reverse | TTTACCCCACCGTCAGTGATC |
| **NOD2** | Forward | GCCACGGTGAAAGCGAAT |
|  | Reverse | GGAAGCGAGACTGAGCAGACA |
| **RIG-I** | Forward | AGGAAAACTGGCCCAAAACT |
|  | Reverse | TTTCCCCTTTTGTGGTTGTG |
| **MDA-5** | Forward | GTGCATGGAGGAGGAACTGT |
|  | Reverse | GTTATTCTCCATGCCCCAGA |
| **NLRP1** | Forward | ACTATACTTCCCGAGGCATCCTT |
|  | Reverse | TGGTCTTGGAAGTCAGTGTGAGT |
| **NLRC4** | Forward | CTCTCATGGTGGAAGCCAGTCC |
|  | Reverse | GACAGAGACTTGACTATGTAATCC |
| **IL-1β** | Forward | ATGGCAGAAGTACCTAAGCTCGC |
|  | Reverse | ACACAAATTGCATGGTGAAGTCAGTT |
| **IL-6** | Forward | ATTCGGTACATCCTCGAC |
|  | Reverse | GGGGTGGTTATTGCATC |
| **TNF-α** | Forward | GGCTCCAGGCGGTGCTTGTTC |
|  | Reverse | AGACGGCGATGCGGCTGATG |
| **IL-18** | Forward | ATGGCTGCTGAACCAGTAGAAG |
|  | Reverse | CAGCCATACCTCTAGGCTGGC |
| **TGF-β** | Forward | AAGGCGAAAGCCCTCAATTT |
|  | Reverse | CAGCAACAATTCCTGGCGATA |
